# A new model for predicting intravenous immunoglobin-resistant Kawasaki disease in Chongqing: a retrospective study on 5277 patients

**DOI:** 10.1038/s41598-019-39330-y

**Published:** 2019-02-11

**Authors:** Xu-Hai Tan, Xiao-Wei Zhang, Xiao-Yun Wang, Xiang-Qian He, Chu Fan, Tie-Wei Lyu, Jie Tian

**Affiliations:** 1 Department of Heart Centre, Children’s Hospital of Chongqing Medical University, Ministry of Education Key Laboratory of Child Development and Disorders, Chongqing, 400014 China; 2 Yidu Cloud (Beijing) Technology Co., Ltd., Beijing, 100101 China; 3 College of Medical Informatics, Chongqing Medical University, Chongqing, 400016 China

## Abstract

Accurate evaluation of individual risk of intravenous immunoglobin (IVIG)-resistance is critical for adopting regimens for the first treatment and prevention of coronary artery lesions (CALs) in patients with Kawasaki disease (KD). Methods: The KD patients hospitalized in Chongqing Children’s Hospital, in west China, from October 2007 to December 2017 were retrospectively reviewed. Data were collected and compared between IVIG-resistant group and IVIG-responsive group. The independent risk factors were determined using multivariate regression analysis. A new prediction model was built and compared with the previous models. Results: A total of 5277 subjects were studied and eight independent risk factors were identified including higher red blood cell distribution width (RDW), lower platelet count (PLT), lower percentage of lymphocyte (P-LYM), higher total bile acid (TBA), lower albumin, lower serum sodium level, higher degree of CALs (D-CALs) and younger age. The new predictive model showed an AUC of 0.74, sensitivity of 76% and specificity of 59%. For individual’s risk probability of IVIG-resistance, an equation was given. Conclusions: IVIG-resistance could be predicted by RDW, PLT, P-LYM, TBA, albumin, serum sodium level, D-CALs and age. The new model appeared to be superior to those previous models for KD population in Chongqing city.

## Introduction

Kawasaki disease (KD) is an acute autoimmune systemic vasculitis disease, mainly affecting young children and characterized by bilateral conjunctival inflammation, atypical rash, etc. The most serious consequence of KD is coronary artery lesions (CALs), which is associated with the prognosis of KD^1^. Prompt treatment with high-dose (2 g/kg) intravenous immunoglobulin (IVIG) could significantly reduce manifestations of KD and CALs. However, 10–20% of the KD patients are resistant to IVIG^2,3^. Thus, after initial IVIG administration, recrudescent or persistent fever may occur and further treatment is required at 48 hours after the initial use of IVIG, such as the second administration of IVIG, corticosteroids, etc^4^. The incidence of CALs in IVIG-resistant KD group was significantly higher than that in the IVIG-sensitive KD group (71% versus 5%, p < 0.0001)^5^. Moreover, studies have suggested that IVIG-resistance is an independent risk factor for giant coronary aneurysms^6,7^. Therefore, to early detect the IVIG-resistant KD patients and improve prognosis, it is important to identify the risk possibility of IVIG-resistance and take appropriate regimens early.

The etiology and underlying biology of KD have not been completely elucidated. It is still a challenge for pediatricians to quickly diagnose KD, especially when diagnosing the children with atypical or incomplete KD. Many studies have tried to explore the methods to identify the disease more effectively and accurately. Previous studies reported that C-reactive protein, neutrophils, serum sodium, aspartate aminotransferase (AST), alanine aminotransferase (ALT), albumin, erythrocyte sedimentation rate (ESR), age, etc. are the risk factors of IVIG-resistance^5,8–12^. Based on those risk factors, some prediction models for IVIG-resistant KD were established, including Fukunishi^3^, Egami^8^, Kobayashi^9^ and Sano^5^ scoring system from Japan and Yang^10^ *et al*., Wang^11^ *et al*. and Tang^12^ *et al*. models from China. Those prediction methods, however, have limitations considering they are specific for Japan, North China and East China. As acknowledged, the risk factors of IVIG-resistance are likely to be different in different regions and populations^13–15^. The prediction models developed from the Japanese population may not prove to be accurate and sensitive enough when applied in the Chinese population. For instance, Kobayashi prediction model, which was developed based on a sample of 546 Japanese patients, showed rather unsatisfactory results when applied in Chinese population. The sensitivity and specificity of Kobayashi model were reported as 86.0% and 67.0% respectively when applied in the Japanese population while as 48.8% and 71.6% when applied in 1177 Chinese KD patients^15^. Although there are prediction models based on KD population in east China and north China, we still lack a prediction model specific for the population in Chongqing city, one of the biggest cities in western China, considering the very large area of China.

In this study, we retrospectively reviewed 5277 KD patients from Chongqing, trying to identify risk factors and establish a new prediction model for IVIG-resistance in Chongqing city. The predictive ability, sensibility and specificity of our new model were further compared with the previously established models including Egami^8^, Kobayashi^9^ and Sano^5^ scoring system from Japan and the model established by Yang^10^ *et al*. from China

## Materials and Methods

### Patients

The KD patients who were hospitalized in Chongqing Children’s Hospital from October 2007 to December 2017 with discharge diagnosis of KD were enrolled into the study. According to the diagnostic guidelines of Kawasaki Disease Version 5^16^, the diagnosis criteria were at least 5 days of fever accompanied by 4 or 5 items of the following clinical manifestations: bilateral conjunctival injection, changes in the lips and oral cavity, non-purulent cervical lymphadenopathy, polymorphous exanthema and changes in the extremities. The presence of three or fewer of the above manifestations was defined as incomplete KD. The inclusion criterion was KD as main diagnosis when the patients were first discharged. The exclusion criteria were incomplete KD and other diseases which are easily confused with KD, such as toddler’s idiopathic arthritis; those patients were also excluded who had been given IVIG treatment in other medical institutions before admission and who didn’t receive IVIG treatment during hospitalization.

### Definition and data collection

IVIG-resistant KD was defined as the KD patients with a persistent or recurrence of fever >37.3 °C at any time during 48 hours to two weeks after initial IVIG treatment, and accompanied by one or more of the main symptoms^15^. The presence of coronary artery lesion was defined as coronary artery diameter ≥2.5 mm in patients aged 0–3 years old, ≥3.0 mm in patients aged 3–9 years old and ≥3.5 mm in patients older than 9 years old^17^. As for the degree of CALs (D-CALs), localized dilatation with internal diameter ≤4 mm, the dilatation with the internal diameter between 4 mm and 8 mm, and the dilatation with the internal diameter ≥8 mm were defined as slight CALs, moderate CALs and severe CALs respectively^18^. The patients were also classified according to age, that was age ≤6 months and age >6 months^8,12^.

All demographic characteristics, imaging data and the laboratory data prior to the initial use of IVIG were collected. The demographic characteristics included age (month), sex, total cost and in-hospital time; imaging data prior to the initial use of IVIG included presence of coronary artery lesions and degree of CALs. The laboratory data included red blood cell(RBC), absolute value of red blood cell distribution (RDWa), red blood cell distribution width (RDW), packed cell volume, erythrocyte morphology, mean platelet volume (MPV), platelet distribution width (PDW), thrombocytocrit, platelet count (PLT), white blood cell (WBC), leucocyte morphology, mean corpuscular hemoglobin (MCH), lymphocyte count, percentage of lymphocyte (P-LYM), neutrophil count, percentage of neutrophil, monocyte, platelet-large-cell ratio (P-LCR), hemoglobin (HB), lymphocyte/neutrophil(LNR), urinary bile proto, leucocyte morphology, hematuria, urine specific gravity (low/normal/high), phagocyte, urine protein, white blood cell (stool), urobilirubin, gamma-glutamyl transpeptidase(GGT), alanine transaminase (ALT), aspartate aminotransferase (AST), lactic dehydrogenase(LDH), alkaline phosphatase (ALP), AST/ALT, total bile acid (TBA), direct bilirubin (DBIL), total bilirubin (TBIL), total protein (TP), albumin, prealbumin, globulin, creatinine, blood urea nitrogen (BUN), ketone body (KET), uric acid (UA), C-reactive protein (CRP), erythrocyte sedimentation rate (ESR), serum inorganic phosphorus, serum sodium, serum potassium, serum magnesium, serum chlorine and serum calcium. If there were more than two laboratory reports concerning CRP, blood-routine, kidney function, urine-routine, electrolytes and liver function, we used the reports with the greatest value of CRP, neutrophils ratio, urea nitrogen, urinary protein and lowest albumin and sodium ion concentration. All those laboratory variables were routinely obtained in our clinical practice.

For using the previous models, we kept the predictors in those models and re-estimate the coefficients with the current data set to build the models. The predictive ability of the new model was compared with the previous models including Kobayashi^9^, Egami^8^ and Sano^5^ scoring systems from Japan and Yang prediction model^10^ from China.

### Statistical analysis

All data were presented as count with percentage for categorical variables and mean ± standard deviation (SD) for continuous variables. For the variables with miss rate <25%, multiple imputation was used. The Mann-Whitney U test was used for the comparison of the intergroup continuous variables; the Chi-square test was used for the comparison of categorical variables between the two groups. P < 0.05 was considered statistically significant. The selected variables significantly different between groups entered into the multivariate analyses. For building the prediction model of IVIG-resistant KD, 70% of the patients were randomly selected from the whole sample, including the IVIG–resistant KD and IVIG-responders, by generating random list of number; the other 30% of the patients’ data were used for testing the new model. To determine independent predictors of IVIG resistance, multivariate logistic regression analysis with least absolute shrinkage and selection operator (LASSO) was performed using the indicators with significant difference derived from the univariate analysis; the OR and 95% CI were calculated. The OR value was used to determine the score of an independent risk factor and build the new prediction model. Hosmer-Lemeshow goodness of fit (GOF) test was used to test the model, and p > 0.05 indicated that the prediction model fit the sample data. Receiver operating characteristic (ROC) curve and the area under the curve (AUC) were used to determine the predictive ability, sensitivity and specificity of the prediction model. To identify personal risk probability of IVIG-resistance that could be used in the nomogram, an equation was given.

Data analysis was conducted using R Project for Statistical Computing (R version 3.4.1).

### Ethic statement

The present study protocol was reviewed and approved by the Ethics Committee of the Children’s Hospital Affiliated to Chongqing Medical University, and with the its approval, this study required no conformed consent. All methods were performed in accordance with Declaration of Helsinki and the relevant guidelines.

## Results

### Sample collection

A total of 5277 subjects met the inclusion criteria and were enrolled into the study, including 348 cases of IVIG resistance (348/5277, 6.59%) and 4929 cases of IVIG responder (4929/5277, 93.41%). Fifty-seven variables were collected, including 4 demographic variables, 1 imaging variable and 52 laboratory variables. The variable, unconjugated bilirubin, was excluded due to its missing rate of 58%.

### Comparison between IVIG-resistant KD and IVIG-responsive KD by univariate analysis

According to univariate analysis (Table 1), 24 variables were significantly higher in the IVIG-resistant group than in the IVIG-responsive group, including RDWa, RDW, erythrocyte morphology, MPV, PDW, Neutrophil count, Percentage of neutrophil, P-LCR, GGT, ALT, AST, lactic dehydrogenase, TBA, DBIL, TBIL, creatinine, BUN, UA, urine protein (positive), leucocyte morphology (urine, positive), urobilirubin (positive), white blood cell (stool, positive), CRP and D-CALs; 18 items were significantly lower in the IVIG-resistant group including RBC, PCV, thrombocytocrit, PLT, lymphocyte, HB, P-LYM, LNR, AST/ALT, TP, albumin, PALB, serum inorganic phosphorus, serum sodium, serum potassium, serum magnesium, serum calcium and age. Besides, the total cost and in-hospital time were significantly higher in IVIG-resistant group, indicating higher burden in IVIG-resistant KD patients.

**Table 1:** Univariate analysis comparison of clinical and laboratory indexes in IVIG responsive and resistant patients.

Variable	IVIG responsive		IVIG resistant		*P*-value
	N	Mean ± SD/Counts (%)	N	Mean ± SD/Counts (%)	
**Blood test**					
Red blood cell count, 1012/L	4014	3.98 ± 0.44	306	3.89 ± 0.47	0.001
Absolute value of Red blood cell Distribution, fL	3727	40.30 ± 4.34	287	41.12 ± 4.73	0.002
Red blood cell distribution width, %	3990	13.89 ± 1.68	301	14.30 ± 2.13	<0.001
Packed cell volume, %	4013	31.99 ± 3.38	306	31.09 ± 3.70	<0.001
Erythrocyte morphology (normal/abnormal)*	3815	245 (0.06)	298	30 (0.10)	0.021
Mean platelet volume, fL	3777	9.90 ± 1.08	292	10.11 ± 1.21	0.004
Platelet distribution width, fL	3882	11.48 ± 2.19	297	11.87 ± 2.50	0.002
Thrombocytocrit, %	3715	0.45 ± 0.56	280	0.38 ± 0.52	<0.001
Platelet count, 10^9^/L	4014	384.18 ± 155.44	306	338.13 ± 164.52	<0.001
White blood cell, 10^9^/L	4013	15.31 ± 6.24	306	15.53 ± 6.40	0.626
Mean Corpuscular Hemoglobin, pg	3846	26.28 ± 2.10	299	26.14 ± 2.16	0.206
Lymphocyte count, 10^9^/L	3760	3.70 ± 2.02	293	2.97 ± 2.09	<0.001
Percentage of lymphocyte	4014	0.26 ± 14	306	0.2 ± 0.13	<0.001
Neutrophil count, 10^9^/L	3927	10.84 ± 5.63	300	11.75 ± 5.65	0.004
Percentage of neutrophil	4014	0.69 ± 0.15	306	0.75 ± 0.15	<0.001
Monocyte count, 10^9^/L	3686	0.42 ± 0.30	285	0.42 ± 0.35	0.258
Platelet-large-cell ratio, %	3641	24.15 ± 8.15	282	25.78 ± 8.66	0.001
Hemoglobin, g/l	4014	104.26 ± 11.28	306	101.26 ± 12.05	<0.001
Lymphocyte/neutrophil,	3757	0.47 ± 0.51	293	0.35 ± 0.42	<0.001
**Urine test**					
Urinary bile proto (positive)*	4536	100 (0.02)	329	12 (0.04)	0.135
Leucocyte morphology (positive)*	3958	42 (0.01)	301	8 (0.03)	0.028
Hematuria (positive)*	4536	282 (0.06)	329	22 (0.07)	0.824
Proportion (normal/high)*	4536	3014/200 (0.66/0.044)	329	211/23 (0.64/0.07)	0.095
Phagocyte (positive)*	4601	1 (0.00)	330	1 (0.00)	0.300
Urine protein (positive)*	4536	482 (0.11)	329	71 (0.22)	<0.001
Urobilirubin (positive)*	4536	97 (0.02)	329	31 (0.09)	<0.001
**Stool test**					
White blood cell (positive)*	4601	398 (0.09)	330	40 (0.12)	0.041
**Biochemical test**					
Glutamyltranspeptidase, U/L	4550	84.47 ± 110.83	312	118.00 ± 121.52	<0.001
Alanine transaminase, IU/L	4550	68.13 ± 99.16	312	90.08 ± 117.16	<0.001
Aspartate aminotransferase, IU/L	4735	49.44 ± 90.97	336	63.59 ± 83.64	<0.001
Lactic dehydrogenase, IU/L	4736	298.33 ± 153.10	336	316.63 ± 127.08	0.007
Alkaline phosphatase, IU/L	4550	182.97 ± 127.32	312	184.18 ± 92.16	0.332
AST/ALT	4550	1.17 ± 0.83	312	1.05 ± 0.78	0.006
Total bile acid, umol/L	3816	20.56 ± 40.34	247	48.72 ± 77.35	<0.001
Direct bilirubin, umol/L	4187	5.22 ± 10.03	280	10.94 ± 17.53	<0.001
Total bilirubin, umol/L	4545	10.20 ± 13.08	312	18.92 ± 25.03	<0.001
Total Protein, g/L	4550	59.78 ± 6.84	312	58.32 ± 9.24	<0.001
Albumin, g/L	4550	36.92 ± 4.76	312	34.16 ± 5.94	<0.001
Prealbumin, mg/L	3805	64.00 ± 38.47	244	54.85 ± 39.58	<0.001
Globulin, g/L	4550	22.86 ± 5.23	312	24.16 ± 7.97	0.375
Creatinine, umol/L	4425	25.91 ± 15.46	303	29.75 ± 22.54	0.004
Blood urea nitrogen, mmol/L	4424	2.81 ± 1.36	302	3.50 ± 2.55	<0.001
Ketone body*,	4536	0.50 ± 0.99	329	0.43 ± 0.91	0.469
Uric acid, umol	4423	208.08 ± 81.64	303	224.13 ± 100.27	0.036
**Inflammatory factor**					
C-reactive protein, mg/L	3869	60.78 ± 52.32	296	73.28 ± 56.73	<0.001
Erythrocyte sedimentation rate, mm/L	4403	66.50 ± 32.32	322	63.33 ± 32.82	0.074
**Ion**					
Serum inorganic phosphorus, mmol/L	4394	1.31 ± 0.29	306	1.24 ± 0.33	<0.001
Serum sodium, mmol/L	4397	137.26 ± 3.17	306	135.90 ± 3.95	<0.001
Serum potassium, mmol/L	4397	4.23 ± 0.66	306	4.06 ± 0.77	<0.001
Serum magnesium, mmol/L	4395	0.92 ± 0.11	306	0.89 ± 0.12	<0.001
Serum chlorine, mmol/L	4395	101.18 ± 3.73	306	100.84 ± 3.93	0.350
Serum calcium, mmol/L	4108	2.29 ± 0.16	277	2.23 ± 0.16	<0.001
**Imaging**					
Degree of coronary artery lesions (slight/moderate/severe)*	3745	1497/212/24 (0.40/0.06/0.01)	260	135/39/3 (0.52/0.15/0.01)	<0.001
**Demographics**					
Age, month	4928	31.80 ± 24.32	348	29.69 ± 25.68	0.016
Sex (female)*	4928	1893 (0.38)	348	116 (0.33)	0.067
Total costs, RMB	4929	11450.25 ± 3646.82	348	19637.15 ± 9207.81	<0.001
In-hospital time, day	4929	7.65 ± 2.75	348	13.33 ± 6.48	<0.001

ALT: Alanine transaminase; AST: Aspartate aminotransferase; *for categorical variables; N: number of sample; SD: standard deviation; W value for Wilcoxon-Mann-Whitney test; χ2 value for chi-square test.

### Analysis of independent risk factors and establishment of predicting model

For multiple logistic regression analysis, the variables with statistical significance derived from univariate analysis were further selected by LASSO constraints in order to find the optimal value of lambda by balancing accuracy and simplicity. The result suggested that the *log* of the optimal value of lambda was eleven. Among the eleven variables, eight indicators presented statistical significance and were used for multivariate logistic regression analysis (Table 2). The independent risk factors for IVIG-resistant KD were higher RDW, lower platelet count, lower P-LYM, higher TBA, lower albumin, lower serum sodium level, higher degree of CALs and younger age. The OR values (95%Cl) of those risk factors were listed in Table 2.

**Table 2:** The OR (odds ratio) values of the independent risk factors for IVIG-resistant Kawasaki disease.

Risk factors	Multiple logistic regression analysis after LASSO		Multiple logistic regression analysis using the nine indicators with statistical significance	
	OR value (95% confidence interval)	*P*-value	OR value (95% confidence interval)	*P*-value
RDW	1.181 (1.099–1.266)	<0.001	1.189 (1.106–1.274)	<0.001
PLT	0.999 (0.998–1.000)	0.048	0.999 (0.998–1.000)	0.013
P-LYM	0.066 (0.017–0.246)	<0.001	0.052 (0.013–0.193)	<0.001
TBA	1.004 (1.002–1.007)	0.001	1.006 (1.003–1.008)	<0.001
Na	0.954 (0.914–0.997)	0.034	0.946 (0.907–0.988)	0.011
Albumin	0.942 (0.916–0.968)	<0.001	0.940 (0.915–0.967)	<0.001
D-CALs1	2.255 (1.654–3.107)	<0.001	2.197 (1.616–3.019)	<0.001
D-CALs2	2.703 (1.550–4.587)	<0.001	2.815 (1.630–4.738)	<0.001
D-CALs3	5.085 (1.099–17.078)	0.017	5.696 (1.234–19.078)	0.010
BUN	1.071 (0.996–1.149)	0.057	/	/
Urobilirubin	1.444 (0.727–2.804)	0.285	/	/
Urine protein	1.385 (0.929–2.027)	0.101	/	/
Age	0.462 (0.302–0.726)	0.001	0.478 (0.313–0.750)	0.001

LASSO, least absolute shrinkage and selection operator; RDW, red blood cell distribution width; PLT, lower platelet count; P-LYM, percentage of lymphocyte; TBA, total bile acid; Na, serum sodium level; D-CALs1, slight degree of coronary artery lesions; D-CALs2, moderate degree of coronary artery lesions; D-CALs3, severe degree of coronary artery lesions; BUN, blood urea nitrogen.

**Figure 1:**
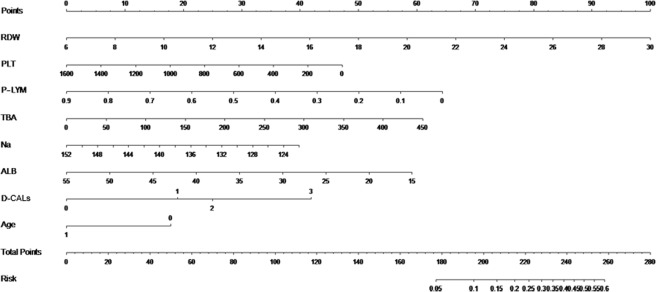
The nomogram for personal risk probability of intravenous immunoglobin-resistant Kawasaki disease. As for age, the patients were classified as age ≤6 months and age >6 months. The risk score represents probability of intravenous immunoglobin-resistance. RDW, RBC; PLT, platelet count; P-LYM, percentage of lymphocyte; TBA, total bile acid; ALB, albumin; Na, serum sodium; D-CALs, degree of coronary artery lesions.

Based on the above result, a nomogram was derived for personal risk probability of IVIG-resistance (Fig. 1). The underlying logistic model is given by the following equation:

$$\begin{array}{c}Log-odds\,of\,having\,IVIG\,resistence=5.772\,+0.173\times RDW+(-0.001)\times PLT\\ \,+\,(-2.966)\times P-LYM+0.006\times TBA+(-0.055)\times Na+(-0.061)\times Albumin\,\\ \,+0.787\times D-CALs1+1.035\times D-CALs2+1.740\times D-CALs3+(-0.738)\times Age\end{array}$$

Thus, individual risk probability of IVIG-resistance could be identified. The coefficients indicate the contribution of the variables. Take RDW for an example, when the other variables are fixed, the odds ratio of having IVIG resistance increases by 18.9% (exp(0.173) − 1 = 1.189 − 1 = 0.189) with one unit increase in RDW. The increase unit in PLT, P-LYM, Na, albumin and age with negative coefficients, would decrease the odds ratio of IVIG-resistant KD; the increase of RDW, TBA, GLB and D-CALs with positive coefficients would increase the likelihood of having IVIG-resistance. The McFadden’s R squared was 0.1223 for this model.

**Figure 2:**
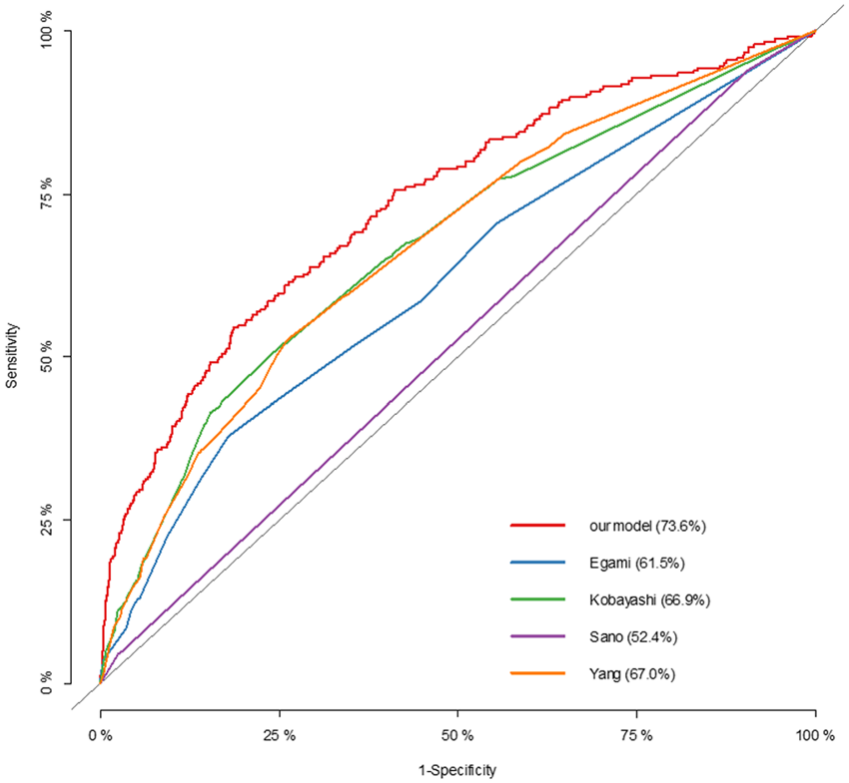
ROC and AUC of the prediction models for IVIG-resistance. The new predictive model for IVIG-resistance showed an AUC of 0.74, Compared with previous IVIG-resistant scoring systems, the new model presented a higher AUC value than the Kobayashi (AUC = 0.68), Egami (AUC = 0.65), Sano (AUC = 0.55) and Yang (AUC = 0.67) methods. ROC, receiver-operator characteristic curves; AUC, area under the curve.

**Figure 3:**
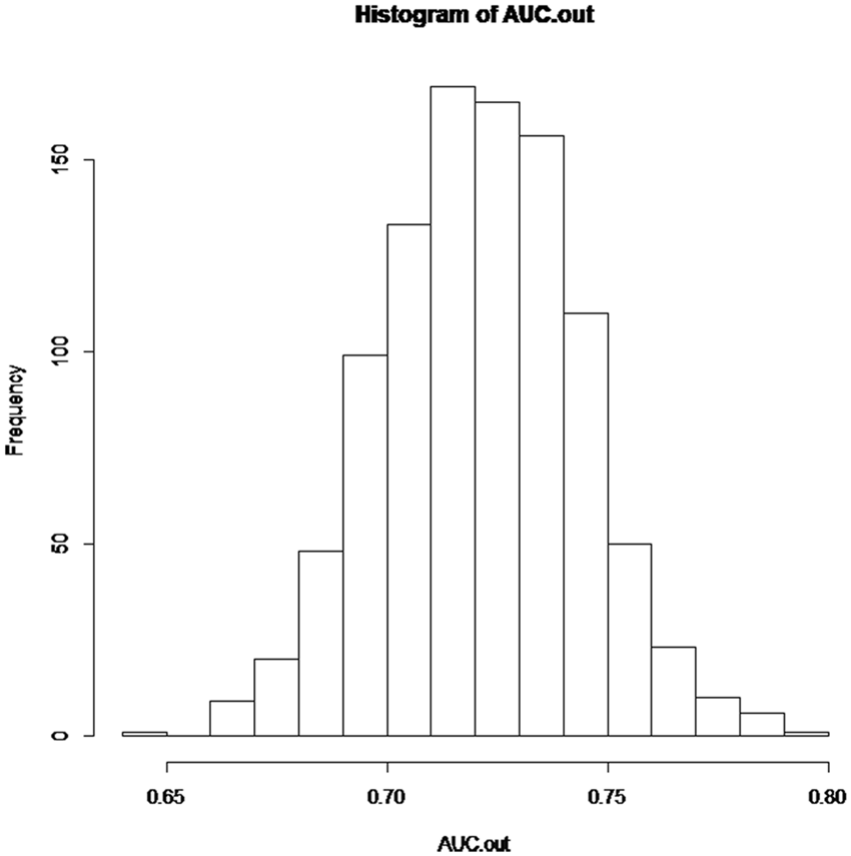
AUCs of the new model by multiple testing. The AUC values of the new model tested by using the randomly-selected 30% of the whole subjects. The AUC on average was 0.72 (range 0.65–0.80).

For model testing, 30% of the total cohort were applied to the new prediction model. GOF test indicated that the prediction model fit the sample data (χ^2^ = 2.3227, p = 0.508). The new predictive model for IVIG-resistance showed an AUC of 0.74 (Fig. 2), sensitivity of 76% and specificity of 59%. Multiple testing was performed to further evaluate the validity of the new prediction model, and the AUCs were shown in Fig. 3. The AUC on average was 0.72 (range 0.65–0.80), indicating the value of AUC was valid.

### Comparison between the new-established model and the previous scoring systems

Compared with previous IVIG-resistant scoring systems, the new model (AUC = 0.74) presented a higher AUC value than the Kobayashi (AUC = 0.68), Egami (AUC = 0.65), Sano (AUC = 0.55) and Yang (AUC = 0.67) methods. Those previous scoring systems were applied to the cohort in this study, and the result showed that sensitivity and specificity of the new prediction model were better than those previous scoring systems (Table 3).

**Table 3:** The predictive ability of the new model and the previous models.

	The new model	Kobayashi model	Egami model	Sano model	Yang model
Sensitivity	0.76	0.75	0.72	0.95	0.67
(95% CI)	(0.70–0.81)	(0.69–0.80)	(0.66–0.77)	(0.92–0.98)	(0.61–0.73)
Specificity	0.59	0.48	0.44	0.1	0.57
(95% CI)	(0.57–0.60)	(0.47–0.50)	(0.42–0.46)	(0.09–0.11)	(0.55–0.58)

AUC, area under the curve; CI, confidence interval.

## Discussion

Currently, the treatment of KD mainly depends on high dose of IVIG, however, IVIG-resistant KD is not sensitive to IVIG and additional treatment cannot quickly and effectively reduce vascular inflammation after the initial use of IVIG or after the diagnosis of IVIG-resistance^19–21^. Thus, it results in increased incidence of CALs, which is harmful to the KD prognosis. It would possibly reduce the CALs incidence in the IVIG-resistant KD patients if additional treatment is adopted early before the initial use of IVIG^5^. Therefore, there is an urgent need to build a prediction model for IVIG-resistant KD with high predictive ability for specific populations in different areas. Here, we reviewed 5277 KD patients from Chongqing city, in West China, and built a new prediction model which appeared to be superior to those previous models when applied in this KD population.

In this study, the percentage of IVIG-resistance was 6.25%, which was far below the percentage of 10%-20% in the studies of Fukunishi^3^ *et al*. and Sleeper^2^ *et al*. and was close to the percentage of 5.1% in Tang’s study^12^ and 5.0% in Qian’s^22^. This might be attributed to the different study populations, the bigger sample size of this study, and the definition of IVIG-resistance. In Sano’s study, IVIG-resistance was defined as persistent fever >24 hours after the completion of initial IVIG infusion while in our study it’s defined as persistent fever >48 hours and duration of initial IVIG use ≥5 days. The initial incidence of CALs judged by absolute diameter was 68% in IVIG-resistant group and 47% in IVIG-responsive group in this study. The initial CALs incidence in IVIG-resistant group was close to Han’s study^23^ and was much higher than Chantasiriwan’s study^24^, but the incidences in IVIG-responsive groups were similar to theirs.

There were several prediction models for IVIG-resistant KD. The risk factors in those models include age of month <6; IVIG treatment within 4 days of illness; abnormal first echocardiographic results; higher levels of CRP, ALT, AST, PCT, neutrophil ratio, percentage of band cell, TBIL and LDH; and lower levels of PLT, serum sodium, hemoglobin and pericardial effusion, etc^9,11,12,15,25–27^. However, those models could not present high predictive ability in populations from different regions. The independent risk factors reported in the previous prediction model, such as CRP, AST, ALT, TBL, NEU%, ALB, GGT, LDH and LNR were significantly different between IVIG-responsive and IVIG-resistant group in our study, but they failed to enter in the final logistic regression model^3,5,8–11^. Besides, the results of univariate analysis may be different in different populations between IVIG-responsive and IVIG-resistant group. For instance, GGT level was significantly different between the two groups in our and Wang’s study, while wasn’t in Yang’s and Kobayashi’s study. Serum chlorine level was significantly different between the IVIG-responders and the IVIG-resistant in Kobayashi’s study, while was not in our and Wang’s study. AST was an independent risk factor in Kobayashi’s and Sano’s study and ALT was an independent risk factor in Egami’s study, but those two factors didn’t show statistical difference in the univariate analysis in Yang’s study^5,8–11^. It might be attributed to that KD pathology is related with genetic polymorphisms, and the reported genetic determinants of KD were different in various populations^28^. The genetic polymorphisms and unknown etiology might make the risk factors of IVIG-resistant different in different populations.

We expected to identify new risk factors of IVIG-resistant KD and establish a more accurate prediction model for Chongqing city. Therefore, this study collected demographic, imaging and laboratory information from 5277 KD patients as completely as possible. The sample size and the variables included in our study were much larger than previous IVIG-resistant KD prediction models. In the present study, a total of 57 variables were successfully collected and included in the univariate analysis, of which 42 factors showed significant difference between the two groups. Eight independent risk factors were identified, among which RDW, P-LYM and D-CALs were not identified as predictive indicators for IVIG-resistance in previous studies. We also found that some new factors were significantly different between the two groups, including PCV, MPV, PDW, thrombocytocrit, P-LCR, lymphocyte/neutrophil, urine protein, urobilirubin, AST/ALT, PALB, serum inorganic phosphorus, serum magnesium and serum calcium. But those were not independent risk factors. In this study, we didn’t include the patients who received initial IVIG treatment within 4 days of illness because the diagnosis criteria of Kawasaki disease required fever duration ≥5 days.

Several studies reported that the platelet counts decreased in IVIG-resistant KD patients^9,11,29^. In our study, we found platelet changed in morphology in addition to the decreased counts in IVIG-resistance group. The reduction of platelets might be associated with CAA-induced platelet consumption, which was followed by a compensatory change in platelet morphology, with an increased volume of platelet and a larger PDW. Urinary protein was higher in IVIG-resistance group than in IVIG-responsive group, which might imply a more severe glomerular vasculitis and increased glomerular vascular permeability in IVIG-resistant KD patients. Besides the increase of ALT, AST, TBIL and LDH, we also observed higher urobilirubin and lower PALB in IVIG-resistant KD group, which suggested that the patients with IVIG-resistance might have more severe systemic inflammation and vasculitis in liver^5^.

As stated in the 5-minute Pediatric Consult (Second Edition)^30^, the concentration of serum inorganic phosphorus and serum sodium decreased in patients with KD and low concentration serum potassium was related with CAA^31^. The significantly lower concentration of serum inorganic phosphorus, serum potassium, serum magnesium, serum calcium and serum sodium and higher concentration of BUN were observed in IVIG-resistant group in our study, which indicated that kidney vasculitis might exert negative effect on renal function and tubular reabsorption.

The final risk factors selected to undergo multivariate analysis for predicting IVIG-resistance, including RDW, platelet count, P-LYM, TBA, albumin, serum sodium level, D-CALs and age. Among those variables, TBA, serum sodium, albumin, platelet count and age of month have been reported in previous studies. The increased RDW was related to anemia, which was consistent with Durongpisitkul’s study. The reduced P-LYM represented higher percentage of neutrophil in blood and more severe inflammation, which was also reported in the studies of Durongpisitkul *et al*. and Wang *et al*.^11,32^. The new model for IVIG-resistant KD prediction was generated based on those risk factors, with the AUC of 0.74, sensitivity of 76% and specificity of 59%. The AUC value, sensitivity and specificity in this study showed better accuracy compared with the previous models when applied in the population from Chongqing city.

This study has considered more medical information besides the factors included in the previous prediction models, in order to find the potential hint of the disease. The factors including RDW, D-CALs and P-LYM were rarely mentioned in previous prediction models. In the future, we will conduct a prospective study to further evaluate the effectiveness of this new model. Still, this study has some limitations. Firstly, it’s a retrospective study in a single center. Secondly, multiple clinical teams participated in the care and measurement of the patients. Thirdly, due to the lack of patients’ height data, no mean body-surface-area (BSA) adjusted Z-score was available; we will take it into consideration in our future study. Last, some data items were missing, which might result in bias in statistical analysis; for the variables with miss rate <25%, multiple imputation was done to decrease bias in this study. With the large sample size, we thought we could still draw a relatively valid conclusion.

## Conclusion

The IVIG-resistance could be predicted using the values of RDW, PLT, P-LYM, TBA, ALB, serum sodium level, D-CALs and age. The new model of predicting IVIG-resistant KD appeared to be superior to those previous prediction models for the KD population in Chongqing city. Further study is necessary to validate the utility of this new model.
